# Fascioliasis and Intestinal Parasitoses Affecting Schoolchildren in Atlixco, Puebla State, Mexico: Epidemiology and Treatment with Nitazoxanide

**DOI:** 10.1371/journal.pntd.0002553

**Published:** 2013-11-21

**Authors:** José Lino Zumaquero-Ríos, Jorge Sarracent-Pérez, Raúl Rojas-García, Lázara Rojas-Rivero, Yaneth Martínez-Tovilla, María Adela Valero, Santiago Mas-Coma

**Affiliations:** 1 Laboratorio de Parasitología y Vectores, Facultad de Biología, Benemérita Universidad Autónoma de Puebla, Ciudad Universitaria, Col. Jardines de San Manuel, Puebla, Puebla, México; 2 Laboratorio de Parasitología, Instituto de Medicina Tropical “Pedro Kouri”, Ciudad de la Habana, Cuba; 3 Servicio de Pediatría, Hospital Universitario de Puebla, Col. Volcanes, Puebla, Puebla, México; 4 Departamento de Parasitología, Facultad de Farmacia, Universidad de Valencia, Burjassot, Valencia, Spain; McGill University, Canada

## Abstract

**Background:**

The Atlixco municipality, Puebla State, at a mean altitude of 1840 m, was selected for a study of *Fasciola hepatica* infection in schoolchildren in Mexico. This area presents permanent water collections continuously receiving thaw water from Popocatepetl volcano (5426 m altitude) through the community supply channels, conforming an epidemiological scenario similar to those known in hyperendemic areas of Andean countries.

**Methodology and Findings:**

A total of 865 6–14 year-old schoolchildren were analyzed with FasciDIG coproantigen test and Lumbreras rapid sedimentation technique, and quantitatively assessed with Kato-Katz. Fascioliasis prevalences ranged 2.94–13.33% according to localities (mean 5.78%). Intensities were however low (24–384 epg). The association between fascioliasis and the habit of eating raw vegetables was identified, including watercress and radish with pronouncedly higher relative risk than lettuce, corncob, spinach, alfalfa juice, and broccoli. Many *F. hepatica*-infected children were coinfected by other parasites. *Entamoeba histolytica*/*dispar*, *Giardia intestinalis*, *Blastocystis hominis*, *Hymenolepis nana* and *Ascaris lumbricoides* infection resulted in risk factors for *F. hepatica* infection. Nitazoxanide efficacy against fascioliasis was 94.0% and 100% after first and second treatment courses, respectively. The few children, for whom a second treatment course was needed, were concomitantly infected by moderate ascariasis burdens. Its efficacy was also very high in the treatment of *E. histolytica*/*E. dispar*, *G. intestinalis*, *B. hominis*, *H. nana*, *A. lumbricoides*, *Trichuris trichiura*, and *Enterobius vermicularis*. A second treatment course was needed for all children affected by ancylostomatids.

**Conclusions:**

Fascioliasis prevalences indicate this area to be mesoendemic, with isolated hyperendemic foci. This is the first time that a human fascioliasis endemic area is described in North America. Nitazoxanide appears as an appropriate alternative to triclabendazole, the present drug of choice for chronic fascioliasis. Its wide spectrum efficacy against intestinal protozooses and helminthiasis, usually coinfecting liver fluke infected subjects in human endemic areas, represents an important added value.

## Author Summary

A human fascioliasis endemic area is described for the first time in North America. In Atlixco, Puebla State, Mexico, prevalences of 2.94–13.33% by *F. hepatica* were found in 865 6–14-year-old schoolchildren by FasciDIG coproantigen test and Lumbreras rapid sedimentation technique, and quantitatively assessed with Kato-Katz. Prevalences peaked in the 8–10-year age group. The use of a coproantigen test proved to be useful, as most children (72%) did not shed eggs. The link of fascioliasis risk with consumption of raw vegetables other than watercress suggests contamination when washing terrestrial vegetables with untreated water and/or in plant cultures using natural water for irrigation. The relatively high coinfection percentage with *Giardia intestinalis* and the lack of a treated water supply system reaching all dwellings suggest that infection may also occur by water drinking. Nitazoxanide efficacy against fascioliasis is worth mentioning, becoming an alternative to triclabendazole. Its wide spectrum against intestinal protozooses and helminthiases, usually coinfecting *Fasciola* infected subjects in human endemic areas, represents an important added value. In Atlixco, the low fascioliasis intensities indicate that no special precaution will be needed for child treatment (epg<400). The mesoendemic situation, with hyperendemic foci, adds concern about possible human fascioliasis underestimation in other areas of Mexico.

## Introduction

Fascioliasis is a parasitic disease caused by two liver fluke species: *Fasciola hepatica* of almost global distribution due to the very large presence of their specific lymnaeid snail vectors, and *F. gigantica* only in Africa and Asia due to the distribution of their specific snail vectors restricted to these two continents [1]. The high pathogenicity of this disease is well known in livestock since long ago [2], despite of which it was only considered a secondary disease in humans due to the number of only around 2500 reported cases before the 90 s [3]. The scenario began to change from that decade, due to the description of large endemic areas including even human hyperendemic situations in countries such as Bolivia [4], Peru [5], [6], Egypt [7], Iran [8], [9] and others.

The high prevalences and intensities detected in several human endemic areas, mainly affecting children and females, together with the increase of human reports in numerous countries, conform an emerging scenario. This new worldwide situation is not always understandable by only considering the higher performance of the present diagnostic techniques, even taking into account up to which level this disease was in need for an update even on fundamental aspects such as the etiological coprological diagnostics in both human and animals [10]. The origin of fascioliasis emergence in recent years has been argued to be related to climate change, at least in part and in given countries [11], as a consequence of the high dependence of fascioliasis transmission on climate and environmental characteristics [12], [13]. This new worrying scenario has forced many multidisciplinary studies, including results showing old misunderstandings due to the simple extrapolation of knowledge about animal fascioliasis to human fascioliasis [1], [10], [14].

The increasing importance of human fascioliasis additionally relies on the results of pathogenicity and immunity studies, according to which this disease appears to be pronouncedly more complicated and with a greater impact in long-term infection than what was believed until the 90 s [15]–[18]. Emergence, long-term pathogenicity and immunological interactions are in the background of the decision taken by WHO to include this disease within the so-called neglected tropical diseases (NTDs), a group of chronic, debilitating, and poverty-promoting, which are among the most common causes of illness of the poorest people living in developing countries. Their control and elimination is now recognized as a priority for achieving United Nations Millennium Development Goals and targets for sustainable poverty reduction [19], [20].

In the Americas, fascioliasis is caused by only *F. hepatica* mainly transmitted by lymnaeid snail vectors of the *Galba*/*Fossaria* group [21] and distributed throughout, from Canada in the North up to Chile and Argentina in the South [1]. Although human fascioliasis cases have been reported from many Latin American countries, areas of high human impact described in the last two decades focus on Andean countries, mainly in high altitude areas where fascioliasis transmission is increased as a consequence of the adaptation of both liver fluke and lymnaeid vectors to the extreme environmental conditions [22]. Contrary to South America, no serious public health situation due to human fascioliasis has been reported in Central America and North America so far.

In Mexico, human infection by *Fasciola* was noted to be sporadic, with only up to fifty cases reported until the second half of the 20th century [23] including the first human cases described in Atlitxco, Puebla State [24]. Human cases were reported from the states of México, Veracruz, Tabasco, Chiapas Hidalgo, Morelos, Oaxaca, San Luis Potosi and Sinaloa, with the state of Puebla as the one in which human reports were more frequent [23]. Unfortunately, the question is posed on the real situation of human fascioliasis in Mexico nowadays, due to (i) the fact that this disease is of no obligatory declaration, (ii) the lack of appropriate surveys in areas of poverty and high animal infection, and (iii) the well-known fact of rural children not usuall attending hospitals for diagnosis.

The aim of the present study is to report relatively high infection prevalences by *F. hepatica* obtained in a survey of schoolchildren in localities of the Atlitxco municipality. The area selected present characteristics a priori favorable for human infection, including livestock infection, presence of lymnaeids belonging to *Galba*/*Fossaria*, altitude and adequate freshwater collections for transmission. Objectives were the epidemiological description of the fascioliasis situation, assess potential food and drinking infection sources in the area, analyze coinfections of fascioliasis with other intestinal parasitic infections, ascertain the impact of fascioliasis on child nutritional status, and verify the usefulness of nitazoxanide for human chronic fascioliasis treatment as an alternative to the actual drug of choice, triclabendazole for human use, unfortunately not easily available everywhere in Latin America. Results obtained enable us to describe a human fascioliasis endemic area in the New World for the first time outside South America, and pose an important question mark about the numerous rural areas of Mexico presenting similar socio-economic, climatic and environmental characteristics.

## Materials and Methods

### Ethics statement

This study was approved by the Comisión de Bioética of the Hospital Universitario de la Benemérita Universidad Autónoma de Puebla, Puebla, Mexico. Samples from children were obtained after consent from the children's parents, following the principles expressed in the Declaration of Helsinki. The consent from the parent's of the children was written and informed. Consent was also obtained from the local authorities of the Atlixco municipality, as well as from heads and teachers of the schools. At the end of the survey, all children who were diagnosed to be infected by the liver fluke and/or any other parasite species received appropriate treatment with nitazoxanide.

### Study areas

**Figure 1:**
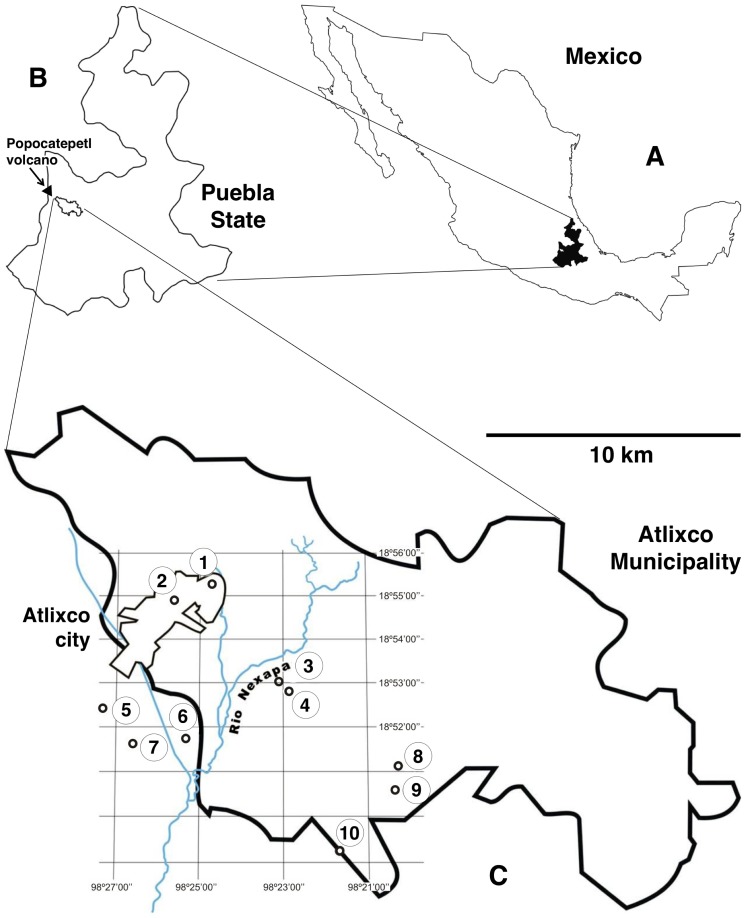
Maps showing the geographical distribution of localities surveyed in Atlixco municipality, Puebla, Mexico. A) Map of Mexico; B) Map of Puebla State; C) Map of Atlixco municipality showing Atlixco city and localities of the schools surveyed: 1 = Tenextepec; 2 = Alta Vista; 3 = Almazán; 4 = Huilotepec; 5 = San Juan Casstillotla; 6 = Juan Uvera; 7 = La Trinidad Tepango; 8 = San Felipe Xonacayucan; 9 = San Jerónimo Caleras; 10 = San Esteban Zoapiltepec.

A total of 865 stool samples from 6–14-year-old children from ten rural schools covering ten different localities of Atlixco municipality were studied: San Esteban Zoapiltepec, San Jerónimo Caleras, Huilotepec, Almazán, Tenextepec, Altavista, San Juan Castillotla, Juan Uvera, La Trinidad Tepango, and San Felipe Xonacayucan. Atlixco municipality (18°49′30″–18°58′30″N; 98°18′24″–98°33′36″W) is located in the State of Puebla, at the centre-south of the country of Mexico (Fig. 1) and has a population of 122,149 inhabitants (2005 census). Atlixco city is at an altitude of 1840 m, similarly as all other localities surveyed.

The area comprising the aforementioned ten localities includes large surfaces devoted to livestock ranching and also free grazing. Sheep and goat breeding for feeding purposes is an important activity in that area. Inhabitants are also known because of watercress consumption and drinking of a beverage made from alfalfa (*Medicato sativa*), a plant raised throughout large surfaces for the feeding of ranched livestock in Chipilo, another locality of the State of Puebla. These alfalfa crop surfaces are irrigated with water from zones presenting *F. hepatica*-infected livestock.

**Figure 2:**
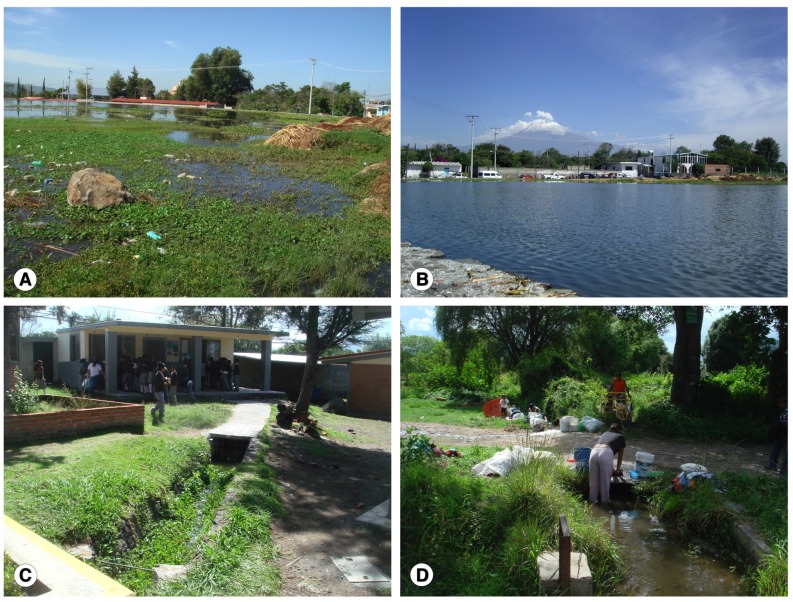
Human fascioliasis transmission and epidemiology in Atlixco municipality, Puebla, Mexico. A) permanent water collection inhabited by freshwater lymnaeid snails, just in front of school (note red school roof in the background); B) permanent water collection inhabited by freshwater lymnaeid snails showing nearby snow-capped Popocatepetl volcano in the background; C) water channel inside school playground with water coming from outside pond illustrated in A; D) women washing in water channel inhabited by lymnaeid vectors, accompanied by small children.

Zones where surveyed schools are located present different kinds of nearby permanent water collections inhabited by high densities of lymnaeid snail species belonging to the *Galba*/*Fossaria* vector group (Gastropoda: Lymnaeidae) [25], [26] (Fig. 2). These natural water collections are continuously receiving thaw water from the relatively close Popocatepetl volcano (the second highest peak in Mexico, with 5426 m altitude, containing glaciers and located only at 22 km northwest from Atlixco city) through the community supply channels. Temperatures around 25–28°C favor lymnaeid population development during many year periods [27].

The surveys were made at random on a given day among all participating students, and the sample size in each school was representative of both the student enrollment in the school and the number of children present on the day of the survey (at least 95%). The 865 children surveyed represent the 93.91% of the total schoolchildren in the aforementioned ten rural schools.

### Stool collection and laboratory methods

The nature of the study was explained to the children, who then were asked to try to pass a small volume of their own faeces into a clean 30-ml plastic, widemouthed, numerated container with snap-on lid. Personal data (name, sex, and age) were taken from school records on delivery of the container. Faecal specimens, one by each student, were transported to the laboratory within the following 6 hours. Three different diagnostic techniques were applied to each stool sample:

the rapid sedimentation procedure [28] was used for a first analysis of the faecal samples, as it has already shown its usefulness for human samples [29]; for protozoans, coloration with eosine and lugol at 1% was applied to sediments and direct smears to facilitate detection and identification; differentiation between *Entamoeba histolytica* and *E. dispar* stages, as well as between eggs of *Necator americanus* and *Ancylostoma duodenale*, respectively most prevalent and relatively rare but anyway widely distributed in Mexico [30], was not made;
a Kato-Katz cellophane faecal thick-smear technique was made following WHO recommendations, using a template delivering about 41.7 mg of faeces [31]. Slides were initially examined within one hour of preparation to avoid over-clarification of some helminth eggs. The Kato-Katz slides were analysed for the egg counts. Intensity of infection, measured as eggs per gram of faeces (epg) was used as an indicator of *F. hepatica* burden in the infected subjects to assess whether special precautions should be taken in the treatment of heavily infected children, according to WHO instructions [32];
the FasciDIG capture ELISA test for fascioliasis diagnosis detects excretory-secretory antigens (ESAs) in faeces [33], [34]. This coproantigen test has already been proved to be useful for the diagnosis of human fascioliasis [35], [36]. Technical procedures were made following the commercial protocol. An optical density of ≥0.24 at 492 nm was considered positive.

### Surveys on food and drinking habits

In order to assess the potential liver fluke metacercarial sources for child infection, two questionnaires, including information about (i) vegetable consumption by the children and (ii) water sources at home, were filled by the same pollster person. Answers from 815 schoolchildren (among the 865 from whom the stool samples were afterwards collected) and from 321 parents (living in the ten localities where schoolchildren were surveyed) were obtained, respectively.

Relative risks (RR) were estimated using a multivariate logistic regression [in the context of risk factors, the resulting Exp(B) are estimates of RR]. An RR higher than 1 was considered to be significant. The correlation of RR of having fascioliasis with consumption of the most frequent vegetables within the local diet (broccoli, spinach, lettuce, radish, rice, corncob, flat maize pancake, beans, alfalfa juice, watercress and watercress plus potato pancake were studied by multivariate logistic regression analysis (PASW Statistics 17). Results were considered statistically significant when *P*<0.05. Two models (Models 1–2) were used in the multivariate logistic regression analysis including presence/absence of fascioliasis as dependent variable. Model 1 included consumption of raw vegetables (broccoli, spinach, lettuce, radish, corncob, alfalfa juice and watercress) as independent variables. Model 2 included consumption of cooked vegetables (rice, flat maize pancake, beans and watercress plus potato pancake) as independent variables.

### Child treatment

Children diagnosed as positive to liver fluke infection and/or to intestinal protozoans and helminths were treated with a course of nitazoxanide of 7.5 mg/kg of body weight each 12 hours (in breakfast and dinner) during seven days. This dose was considered adequate to treat the different protozooses and helminthiases found and the low intensities of the latter, according to the instructions of the medicinal product manufacturer. Post-treatment follow-up was made weekly until disappearance of parasite stages in stools, including blood testing focusing on eosinophilia and anaemia, and additionally negativization of coproantigen test results in the case of fascioliasis, for which a second treatment course was needed only in a relatively few cases.

Nitazoxanide is a thiazolide derivative, a pyruvate ferredoxin oxireductase inhibitor with reported efficacy on a broad parasitological spectrum, such as intestinal protozoans and helminths, including fascioliasis. It is given by the oral route with good bioavailability and is well tolerated, with primarily mild gastrointestinal side effects. The US Food and Drug Administration (FDA) approved nitazoxanide in 2002 for the treatment of diarrhea caused by *Cryptosporidium* species and *Giardia intestinalis* in pediatric patients 1–11 years of age, and in 2004 for its use in adults [37]–[40]. This drug has already been successfully used against intestinal protozoan and helminthic infections in Mexico [41]–[43] as well as in many countries [44].

### Data management and statistical analysis

Statistical analyses were done using SPSS version 15.0. For the evaluation of categorical variables, the chi-square test or Fisher's exact test was used. Odds ratio (OR), 95% confidence intervals and P-values were calculated. Subsequently, stepwise conditional logistic regression was used to determine independent potential risk factors associated with *F. hepatica* infection or *F. hepatica* egg presence in faeces. Associations between *F. hepatica* infection and other parasite species were investigated by 2×2 contingency tables, from which the chi-square statistic was calculated. A P value less than 0.05 was considered statistically significant.

## Results

### Infection analyses

Result on liver fluke infection according to gender and age groups are noted in Tables 1 and 2. Prevalences by *F. hepatica* ranged between a minimum of 2.9% found in the locality of Juan Uvera and a maximum of 13.3% in the locality of San Jerónimo Caleras, with a mean prevalence of 5.8% for the whole area. No significant differences according to localities appeared.

**Table 1:** Sex distribution of *Fasciola hepatica* infection detected in the 865 6–14-year-old schoolchildren analyzed from the localities surveyed in Atlitxco municipality, Puebla State, Mexico, according to the different diagnostic techniques applied.

Localities	Schools	Number of subjects studied			Number of positive by FasciDIG test			Number of positive by Lumbreras technique			Number of positive by Kato-Katz technique			Eggs/g of faeces by Kato-Katz
		Boys	Girls	Total	Boys	Girls	Total	Boys	Girls	Total	Boys	Girls	Total	Range values
San Esteban Zoapiltepec	Ignacio Manuel Altamirano	72	33	105	1	3	4	0	2	2	0	2	2	240 epg
San Jerónimo Caleras	Juventino Rosas	38	22	60	5	3	8	1	3	4	1	3	4	120–288 epg
Huilotepec	José María Morelos	42	28	70	1	2	3	0	2	2	0	2	2	48–240 epg
Almazán	Valerio Trujano	32	17	49	3	0	3	0	0	0	0	0	0	0
Tenextepec	Melchor Ocampo	27	26	53	2	0	2	1	0	1	1	0	1	240 epg
Altavista	Ignacio Zaragoza	116	119	235	9	6	15	1	0	4	1	0	1	384 epg
San Juan Castillotla	Simón Bolivar	29	28	57	2	1	3	0	0	0	0	0	0	0
Juan Uvera	Aquiles Serdán	66	36	102	1	2	3	1	0	1	1	0	1	48 epg
La Trinidad Tepango	Alvaro Obregón	31	20	51	3	3	6	2	1	3	2	1	3	48–120 epg
San Felipe Xonacayucan	Aquiles Serdán	28	55	83	1	2	3	0	0	0	0	0	0	0
TOTAL		481	384	865	28	22	50	6	8	14	6	8	14	24–384 epg

**Table 2:** Age distribution of *Fasciola hepatica* infection detected in the 865 6–14-year-old schoolchildren analyzed from the localities surveyed in Atlixco municipality, Puebla State, Mexico.

Localities	San Esteban Zoapiltepec			San Jerónimo Caleras			Huilotepec			Almazán			Tenextepec			Altavista			San Juan Castillotla			Juan Uvera			La Trinidad Tepango			San Felipe Xonacayucan					
Schools	Ignacio Manuel Altamirano			Juventino Rosas			Jose Maria Morelos			Valerio Trujano			Melchor Ocampo			Ignacio Zaragoza			Simón Bolivar			Aquiles Serdán			Alvaro Obregón			Aquiles Serdán			Total		
Age (years)	N	+	%	N	+	%	N	+	%	N	+	%	N	+	%	N	+	%	N	+	%	N	+	%	N	+	%	N	+	%	N	+	%
6	15	1	6.7	6	0	0.0	26	1	3.8	5	0	0.0	7	1	14.3	31	3	9.7	13	0	0.0	18	1	5.6	10	1	10.0	1	1	100	132	9	6.8
7	13	0	0.0	8	0	0.0	24	0	0.0	6	0	0.0	8	0	0.0	31	3	9.7	7	0	0.0	17	2	11.8	7	1	14.3	6	0	0.0	127	6	4.7
8	14	1	7.1	8	3	37.5	20	2	10.0	7	0	0.0	6	0	0.0	24	2	8.3	7	0	0.0	16	0	0.0	9	0	0.0	20	0	0.0	131	8	6.1
9	24	1	4.2	10	2	20.0	−	−	−	6	0	0.0	9	1	11.1	37	3	8.1	5	0	0.0	8	0	0.0	11	2	18.2	26	1	3.8	136	10	7.4
10	24	1	4.2	10	2	20.0	−	−	−	3	1	33.3	8	0	0.0	24	2	8.3	4	0	0.0	6	0	0.0	5	2	40.0	31	1	3.2	115	9	7.8
11	10	0	0.0	5	0	0.0	−	−	−	12	2	16.7	8	0	0.0	34	2	5.9	6	0	0.0	8	0	0.0	4	0	0.0	6	0	0.0	93	4	4.3
12	5	0	0.0	13	1	7.7	−	−	−	10	0	0.0	7	0	0.0	50	0	0.0	11	2	18.2	10	0	0.0	5	0	0.0	12	0	0.0	123	3	2.4
13	−	−	−	−	−	−	−	−	−	−	−	−	−	−	−	3	0	0.0	3	0	0.0	−	−	−	−	−	−	−	−	−	6	0	0.0
14	−	−	−	−	−	−	−	−	−	−	−	−	−	−	−	1	0	0.0	1	1	100	−	−	−	−	−	−	−	−	−	2	1	50.0
TOTAL	105	4	3.8	60	8	13.3	70	3	4.3	49	3	6.1	53	2	3.8	235	15	6.4	57	3	5.3	83	3	3.6	51	6	11.8	102	3	2.9	865	50	5.8

N = total studied; + = total infected; % = prevalence.

A total of 50 children (28 males and 22 females) were found positive to the coproantigen test, whereas only 14 of them (6 males and 8 females) were shedding eggs in stools. Intensities were always low, between 24 and 384 epg (Table 1). Children infected by the liver fluke were more numerous among the 8–10-year age group (n = 27), followed by the 6–7-year age group (n = 15) and finally the 11–14-year age group (n = 8) (Table 2). No significant differences according to gender or age group appeared.

Results from the answers obtained by means of the questionnaire on vegetable consumption habits of the children are shown in Table 3. Among the different foods examined by multivariate logistic regression analysis, several appeared to be significantly associated with *F. hepatica* infection with increased risk. The association between fascioliasis and the habit of eating raw vegetables was identified, including from more to less risk as follows: watercress (RR = 2250.16) and radish (RR = 675.36) with pronouncedly higher relative risk than lettuce (RR = 56.58), corncob (RR = 31.26), spinach (RR = 16.615), alfalfa juice (RR = 9.51), and broccoli (RR = 7.881).

**Table 3:** Results of multivariate logistic regression analysis of vegetable food habits of children, potentially related to fascioliasis transmission and human fascioliasis and regression coefficients [Exp(B) = RR = relative risk] with significance in different models.

Vegetable consumption	Questionnaire positive answers in total children		Questionnaire positive answers in children positive by FasciDIG		Questionnaire positive answers in children shedding *Fasciola* eggs		Model 1		Model 2	
	answers among 815 children	%	answers among 50 children	%	answers among 14 children	%	Coefficient	Significance	Coefficient	Significance
Broccoli	13	1.60	6	12.00	2	14.28	7.881	0.624		
Spinach	18	2.21	4	8.00	0	0	16.615	0.193		
Lettuce	39	4.79	17	34.00	5	35.71	56.58	0.002		
Radish	130	15.95	41	82.00	9	64.28	675.36	<0.001		
Rice	766	93.99	23	46.00	6	42.86			0.005	<0.001
Corncob	340	41.72	42	84.00	14	100	31.26	0.005		
Flat maize pancake	815	100	48	96.00	13	92.86			<0.001	1.000
Beans	757	92.88	50	100	14	100			1.136	0.711
Alfalfa juice	262	32.15	43	86.00	13	92.86	9.51	0.073		
Watercress	97	11.90	47	94.00	13	92.86	2250.16	<0.001		
Watercress plus potato pancake	591	72.52	50	100	14	100			1.162	0.992

Two models (Models 1–2) were used in the multivariate logistic regression analysis including presence/absence of fascioliasis as dependent variable:

- Model 1 including consumption of broccoli, spinach, lettuce, radish, corncob, alfalfa juice and watercress as independent variables.

- Model 2 including consumption of rice, flat maize pancake, beans and watercress & potato pancake as independent variables.

With regard to water sources at home, answers furnished by parents of the same localities referred to aqueduct (48.87%), pond or water ditch (25.00%), well (22.75%), water tank (2.70%), and channel (0.68%). Combined sources (use of two or more water sources in the same house) was noted in 34.46%. Additionally, during field work, children were sometimes observed to bath, defaecate and urinate in different water collections after leaving school.

Additionally to F. hepatica, three protozoan and five helminth species were found: E. histolytica/E. dispar, Giardia intestinalis and Blastocystis hominis, Hymenolepis nana, Ascaris lumbricoides, Trichuris trichiura, Ancylostomatidae spp., and Enterobius vermicularis. Total prevalences of these parasitic infections and respective distributions according to localities and gender are noted in Table 4, and according to age in Table 5. Infections by A. lumbricoides, T. trichiura, E. histolytica/E. dispar, and Giardia intestinalis appeared to be the most prevalent. An expected slight decrease of their prevalences with age is seen, except in A. lumbricoides and T. trichiura which clearly peak at the 8–10-year-old group. Prevalence data of E. vermicularis may be considered underestimations, as anal swabs, the adequate technique for the detection of the eggs of this nematode species, could unfortunately not be used due of its methodological difficulties in field work.

**Table 4:** Prevalences of parasite species detected in the schoolchildren surveyed in the Atlitxco municipality, Puebla, Mexico, according to localities and gender.

Localities	Schools	Gender	*E. histolytica/dispar*	*G. intestinalis*	*B. hominis*	*F. hepatica*	*H. nana*	*A. lumbricoides*	*T. trichiura*	Ancylosto-matidae	*E. vermicularis*
			%	%	%	%	%	%	%	%	%*
San Esteban Zoapiltepec	Ignacio Manuel Altamirano n = 105	Boys	5.6	2.8	0.0	1.4	1.4	12.5	6.9	2.8	1.4
		Girls	6.1	0.0	3.0	9.1	3.0	15.2	0.0	0.0	0.0
		total	5.8	1.9	1.0	3.8	1.9	13.3	4.8	1.9	1.0
San Jerónimo Caleras	Juventino Rosas n = 60	Boys	5.3	7.9	2.6	13.2	2.6	36.8	13.2	0.0	7.9
		Girls	18.2	13.6	4.5	13.6	4.5	27.3	18.2	0.0	0.0
		total	10.0	10.0	3.3	13.3	3.3	33.3	15.0	0.0	5.0
Huilotepec	José María Morelos n = 70	Boys	7.1	7.1	0.0	2.4	0.0	11.9	2.4	7.1	7.1
		Girls	7.1	14.3	0.0	7.1	10.7	21.4	3.6	3.6	3.6
		total	7.1	10.0	0.0	4.3	4.3	15.7	2.9	5.7	5.7
Almazán	Valerio Trujano n = 49	Boys	12.5	0.0	0.0	9.4	3.1	18.8	3.1	0.0	6.3
		Girls	17.6	11.8	0.0	0.0	0.0	11.8	17.6	0.0	0.0
		total	14.3	4.1	0.0	6.1	2.0	16.3	8.2	0.0	4.1
Tenextepec	Melchor Ocampo n = 53	Boys	7.4	14.8	7.4	7.4	0.0	29.6	18.5	7.4	11.1
		Girls	11.5	11.5	0.0	0.0	7.7	26.9	26.9	3.8	7.7
		total	9.4	13.2	3.8	3.8	3.8	28.3	22.6	5.7	9.4
Altavista	Ignacio Zaragoza n = 235	Boys	6.0	3.4	0.9	7.8	0.9	32.8	8.0	0.0	1.7
		Girls	5.9	5.0	0.0	5.0	0.0	18.5	6.7	0.8	2.5
		total	6.0	4.3	0.4	6.4	0.4	25.5	7.3	0.4	2.1
San Juan Castillotla	Simón Boliva n = 57	Boys	24.1	28.6	0.0	6.9	0.0	41.4	24.1	6.9	6.9
		Girls	22.2	25.0	0.0	3.6	0.0	39.3	28.6	3.6	3.6
		total	23.2	26.8	0.0	5.3	0.0	40.4	26.3	5.3	5.3
Juan Uvera	Aquiles Serdán n = 102	Boys	5.6	11.1	0.0	1.5	2.8	41.7	5.6	2.9	2.8
		Girls	7.2	7.2	0.0	5.6	1.4	23.2	8.7	1.4	1.5
		total	6.7	8.6	0.0	2.9	1.9	29.5	7.6	1.9	1.9
La Trinidad Tepango	Alvaro Obregón n = 51	Boys	3.2	9.7	6.5	9.7	9.7	12.9	6.5	3.2	3.2
		Girls	5.0	10.0	5.0	15.0	10.0	5.0	10.5	5.3	0.0
		total	3.9	9.8	5.9	11.8	9.8	9.8	8.0	4.0	2.0
San Felipe Xonacayucan	Aquiles Serdán n = 83	Boys	7.4	3.7	0.0	3.6	0.0	11.1	3.7	0.0	0.0
		Girls	3.8	1.9	0.0	3.6	3.8	17.0	3.8	0.0	1.9
		total	5.0	2.5	0.0	3.6	2.5	15.0	3.8	0.0	1.3
TOTAL	n = 865	Boys	7.6	7.1	1.3	5.8	1.8	25.3	8.5	2.4	4.0
		Girls	8.5	8.0	0.7	5.7	2.9	20.5	9.9	1.4	2.2
		total	8.0	7.5	1.0	5.8	2.3	23.0	9.2	2.0	3.1

* Underestimating data due to the impossibility of using anal swabs for the detection of *E. vermicularis* eggs.

**Table 5:** Prevalences by intestinal protozoa and helminths found in the 865 schoolchildren surveyed in the Atlitxco municipality, Puebla, Mexico, according to age.

Age groups	*E. histolytica/dispar*		*G. intestinalis*		*B. hominis*		*F. hepatica*		*H. nana*		*A. lumbricoides*		*T. trichiura*		Ancylostomidae spp.		*E. vermicularis*	
	No.	%	No.	%	No.	%	No.	%	No.	%	No.	%	No.	%	No.	%	No.*	%*
6 years (n = 132)	16	12.1	18	13.7	1	0.8	9	6.8	3	2.3	28	21.2	5	3.8	8	6.1	4	3.0
7 years (n = 127)	8	6.3	13	10.2	0	0.0	6	4.7	5	3.9	22	17.3	7	5.5	1	0.8	5	4.0
8 years (n = 131)	9	7.0	8	6.1	1	0.8	8	6.1	7	5.3	22	16.8	11	8.5	5	3.8	1	0.8
9 years (n = 136)	11	8.1	9	6.6	1	0.7	10	7.4	2	1.5	40	29.4	15	11.0	2	1.5	3	2.2
10 years (n = 115)	7	6.1	5	4.3	2	1.7	9	7.8	2	1.7	24	20.9	15	13.0	0	0.0	0	0.0
11 years (n = 93)	6	6.5	2	2.2	1	1.1	4	4.3	1	1.1	29	31.2	13	14.0	0	0.0	6	6.5
12 years (n = 123)	12	9.8	7	5.7	3	2.4	3	2.4	0	0.0	31	25.2	13	10.8	1	0.8	8	6.5
13 years (n = 6)	0	0.0	3	50.0	0	0.0	0	0.0	0	0.0	2	33.3	0	0.0	0	0.0	0	0.0
14 years (n = 2)		0.0	0	0.0	0	0.0	1	50.0	0	0.0	1	50.0	0	0.0	0	0.0	0	0.0
6–14 years (n = 865)	69	8.0	65	7.5	9	1.0	50	5.8	20	2.3	199	23.0	79	9.2	17	2.0	27	3.1

* Underestimating data due to the impossibility of using anal swabs for the detection of *E. vermicularis* eggs.

Many *F. hepatica*-infected children were coinfected by other parasites of the above mentioned species, mainly *A. lumbricoides* and *G. intestinalis* (Table 6). Combined coinfections were with up to four different parasites. Only six boys and four girls, among whom three girls shedding liver fluke eggs, showed no coinfection. The presence of *E. histolytica*/*dispar* (OR = 3.110, P = 0.010), *G. intestinalis* (OR = 3.230, P = 0.009), *B. hominis* (OR = 11.296, P = 0.001), *H. nana* (OR = 8.888, P<0.001) and *A. lumbricoides* infection (OR = 3.929, P<0.001) resulted in risk factors for *F. hepatica* infection. However, this was not the case of *T. trichiura* infection (OR 0.517, P = 0.310).

**Table 6:** Prevalences by intestinal protozoa and helminths found in the 50 schoolchildren presenting fascioliasis among the total of children surveyed in the Atlitxco municipality, Puebla, Mexico, according to age.

Age	*E. histolytica*		*G.intestinalis*		*B. hominis*		*H. nana*		*A.lumbricoides*		*T. trichiura*		Ancylostomidae spp.		*E. vermicularis*	
	No.	%	No.	%	No.	%	No.	%	No.	%	No.	%	No.	%	No.*	%*
6 years (n = 9)	2	22.2	2	22.2	1	11.1	0	0.0	7	77.8	1	11.1	2	22.2	2	22.2
7 years (n = 6)	2	33.3	2	33.3	0	0.0	1	16.7	3	50.0	1	16.7	0	0.0	1	16.7
8 years (n = 8)	1	12.5	0	0.0	0	0.0	3	37.5	6	75.0	0	0.0	0	0.0	0	0.0
9 years (n = 10)	3	30.0	2	20.0	1	10.0	0	0.0	4	40.0	0	0.0	1	10.0	1	10.0
10 years (n = 9)	0	0.0	2	22.2	1	11.1	1	11.1	5	55.6	2	22.2	0	0.0	0	0.0
11 years (n = 4)	1	25.0	1	25.0	0	0.0	0	0.0	1	25.0	0	0.0	0	0.0	1	25.0
12 years (n = 3)	0	0.0	1	33.3	1	33.3	0	0.0	0	0.0	0	0.0	2	22.2	0	0.0
13 years (n = 0)	-	-	-	-	-	-	-	-	-	-	-	-	-	-	-	-
14 years (n = 1)	0	0.0	0	0.0	0	0.0	0	0.0	1	100.0	0	0.0	0	0.0	0	0.0
6-14 years (n = 50)	9	18.0	10	20.0	4	8.0	5	10.0	27	54.0	4	8.0	3	6.0	5	10.0

* Underestimating data due to the impossibility of using anal swabs for the detection of *E. vermicularis* eggs.

### Treatments

The post-treatment follow-up was made with faecal examinations by means of the three diagnostic techniques for up to two months. Of the 50 liver fluke infected children, only three (6.0%) were still intermittently shedding a low number of eggs after the first nitazoxanide treatment course (Table 7). None of these three children was anyway showing eggs in stools after the second treatment course administered one month after the first treatment. Interestingly, these three children were concomitantly infected by moderate burdens (moderate epg counts) of ascariasis.

**Table 7:** Prevalences by intestinal protozoa and helminths found in the 865 schoolchildren surveyed in the Atlitxco municipality, Puebla, Mexico, and results obtained in nitazoxanide treatments.

Parasite species	Total positive subjects		Treated subjects followed		Negative after first treatment course		Negative after second treatment course	
	No.	%	No.	%	No.	%	No.	%
**Protozoa:**								
*Entamoeaba histolytica/dispar*	69	8.0	61	88.4	53	86.8	8	100
*Giardia intestinalis*	65	7.5	54	83.0	50	92.6	4	100
*Blastocystis hominis*	9	1.0	3	33.3	3	100	–	–
**Helminths:**								
*Fasciola hepatica*	50	5.8	50	100	47	94.0	3	100
*Hymenolepis nana*	20	2.3	12	60.0	12	100	–	–
*Ascaris lumbricoides*	199	23.0	150	75.3	150	100	–	–
*Trichuris trichiura*	79	9.2	73	92.4	73	100	–	–
*Ancylostomatidae*	17	2.0	13	76.4	0	0	13	100
*Enterobius vermicularis* *	27	3.1	21	77.7	17	80.9	4	100

* Underestimating data due to the impossibility of using anal swabs for the detection of *E. vermicularis* eggs.

Eosinophilia decreased to normal rates after treatment except in 7 children who only showed a long-term slow decrease and other four children who maintained relatively high levels for up to one year. One of the latter four was detected to re-start faecal egg shedding of both *F. hepatica* and *A. lumbricoides*, an increase of eosinophilia up to 37%, and anaemia. Adequately re-treated, the negativity was afterwards again verified in these four children by the three techniques.

Most of the children infected by intestinal protozoans and helminths could also be treated by nitazoxanide (Table 7). In the majority of cases, diagnostic techniques showed the disappearance of parasitic stages in stools two months after first treatment course application. Only a few children infected by *E. histolytica*/*dispar*, *G. intestinalis*, ancylostomids and *E. vermicularis* were in need for a second treatment course for total cleaning.

## Discussion

### Liver fluke infection

The overall prevalence of 5.8% and local prevalences ranging between 2.9% and 13.3% are the highest so far described not only in Mexico but also in the whole Central American region. The absence of significant differences between prevalences according to localities and the absence of any trend according to their geographical distribution, suggest that this endemic area may have homogeneous transmission and epidemiological characteristics. The Atlixco area may thus be catalogued as human fascioliasis mesoendemics [45]. However, the relatively high local prevalences in San Jerónimo Caleras (13.3%) and La Trinidad Tepango (11.8%) adds concern in front of the possibility for hyperendemic local situations. Field studies to assess whether different lymnaeid species and their spatial distribution may be related to the slightly different local prevalences are in the way. The patchy distribution of fascioliasis as the consequence of the distribution of the water-linked populations of lymnaeid vectors is well known in other human endemic areas [12], [46], [47].

The difference in subject positivity when comparing results obtained with the coproantigen test (50 subjects, 5.78%) and the coprological Lumbreras and Kato-Katz egg finding techniques (14 subjects, 1.62%) (Table 1) is too high as to conclude that it is only related to the fact that most children were still in the acute phase when surveyed. Other aspects which may be at the origin of this difference include the known low sensibility of the Lumbreras and Kato-Katz techniques, the difficulty of egg finding in low burden infections, and the intermittence in egg shedding.

The low epg counts (24–384 epg) found suggest that (i) liver fluke burdens are generally low in this area, and (ii) eggs shed by children may be so scarce as to be easily overlooked. This remembers the diagnostic problems usually posed by egg absence in patients in Europe which are solved with serological tests. Additionally, it should be considered that eggs in humans are known to follow a timely irregular appearance in stool samples [48], which in cases of low burdens may be more pronounced and give rise to egg overlooking when only analyzing one or a few microscopic preparations. These low burdens may be welcome, because it means that in general no special precaution will be needed for child treatment in that area due to epg always lower than 400 [32].

Prevalences by the coproantigen test according to gender do not show statistical differences, neither for the whole area nor after localities, although there is a somewhat higher rate in males than in females (56% vs 44%). Interestingly, the sex ratio of positive cases appears opposite by the egg finding techniques of Lumbreras and Kato-Katz (42.8% vs 57.1%) (Table 1). Although the number of children shedding eggs was insufficient as to reach a significant conclusion, this agrees with results obtained in other human endemic areas where females shed more eggs than males [4].

Prevalences by the coproantigen test according to age were 5.79% (15 positive among a total of 259 children studied), 7.06% (27 of 382), and 3.57% (8 of 224) in the 6–7, 8–10, and 11–14 year-old groups. These results are similar to those found in hyperendemic areas of Andean countries, where a peak in the 9–11 year-old age group is typical [4], [5], [49], [50]. In spite of the evidence of the infection decrease in the 12–15 year-old age group, it should be considered that infection still occurs in adult subjects in such endemic areas, although at lower prevalence rates [4], [7], [49], and that fasciolid parasites are able to survive up to 13.5 years in humans [51]. Consequently, appropriate adult surveys in the localities presenting higher prevalences in children should be performed, and hospitals, medical centres and physicians of Atlixco municipality should be ready to diagnose adult patients presenting suspicious symptomatology.

In Atlixco, the first two human fascioliasis cases were already reported five decades ago [24]. Several additional cases of fascioliasis (0.6% of the subjects analyzed in hospitals) and infection by other helminths were described for the same area shortly after [52]. A few cases were sporadically diagnosed by duodenal exploration in the University Hospital of Puebla throughout the period from 1987 to 2004 [53]. Thus, it may be concluded that (i) human infection rates have increased in recent years, (ii) hygienic-health improvements have not been sufficient in the last five decades, and (iii) most of the human cases in Atlixco are overlooked. The majority of infected subjects of rural areas may not go to medical centers for diagnosis and misdiagnosis may sometimes occur due to insufficiently sugestive symptomatology. Both aspects have already been highlighted in other human fascioliasis endemic areas of Bolivia, Peru and Egypt [51]. Although prevalences and intensities are lower and temperatures higher, the Atlixco 1840-m-altitude area presents many epidemiological characteristics similar to those of the human hyperendemic area of the Northern Bolivian Altiplano at 3800–4100 m altitude, where lymnaeids inhabit permanent water collections maintained from thaw of snow and ice accumulated on the Eastern Andean mountain chain throughout the year [11], [12], [46].

In Mexico, although human infection appears also sporadically reported in recent years [54]–[59], various aspects clearly suggest that the disease should be considered a national health concern: (i) human cases have been reported from several states other than Puebla, such as Hidalgo, Mexico, Veracruz, Chiapas, Oaxaca, San Luis-Potosi, Jalisco and Morelos [23]; (ii) massively infected patients have been diagnosed [60]; (iii) children have been found to be infected in surveys in Chiapas [61] and Mexico city [62]; and (iv) several of the patients recently diagnosed in Canada and the United States were in fact immigrants from Mexico [63]–[67]. Prevalences and intensities found in the present study in Atlixco municipality are the highest so far recorded in the country and add thus concern about the possible level of human fascioliasis underestimation countrywide.

With regard to fascioliasis coinfections with other parasites, the effect of chronic infections by *G. intestinalis* and *A. lumbricoides* on the healthy growth, cognitive development, physical fitness, and iron status of children has already been described [68], [69]. The presence of these parasites makes the analysis of fascioliasis impact on the child development difficult. Futures studies with bigger samples are in need for a correct assessment of the liver fluke impact.

### Food and drinking sources for liver fluke infection

Recent studies have shown that there are more human infection sources than the one traditionally noted, through free-living ( = non-parasitic), encysted metacercariae attached to watercress. The most important sources appear to be linked to vegetables and freshwater. With regard to vegetables, the following should be considered: (i) ingestion of wild freshwater plants; (ii) ingestion of cultivated freshwater plants; (iii) ingestion of wild terrestrial plants; (iv) ingestion of cultivated terrestrial plants; and (v) drinking of beverages made from local plants [9], [70]. Regarding freshwater, three different sources should be taken into account: (i) drinking of contaminated water; (ii) ingestion of dishes and soups made with contaminated water; and (iii) washing of kitchen utensils or other objects with contaminated water [70].

Information obtained from the questionnaires indicates that fascioliasis risk is mainly related to consumption of watercress (Table 3). Watercress is traditionally consumed by the children inhabiting the Atlixco area in different ways, mainly as a watercress sandwich with potatoes (all children in the area who mentioned to consume this freshwater plant) and secondarily with different dressing such as lemon, vinegar or any other dressing. Most human reports in the world have been related to watercress. However, the general term watercress includes different aquatic species such as *Nasturtium officinale* (common watercress), *N. silvestris* and *Roripa amphibia* (wild watercress). Wild watercress has been reported as the main source of human infection in areas where fasciolosis in domestic animals is highly endemic [70].

The analysis of questionnaire responses show that, after watercress, fascioliasis risk appears linked to several vegetables which are usually eaten raw, such as radish, lettuce, corncob, and spinach. Despite being terrestrial, the aforementioned local vegetables may increase the infection risk when washed with contaminated water [70] or cultivated using natural water for irrigation in places where water collections are inhabited by lymnaeids [71].

When considering the responses about water sources at home, a potential involvement of all other vegetables frequently consumed may not be ruled out. Additionally, given the lack of a treated water supply system reaching all dwellings, infection through drinking contaminated water, ingestion of dishes and soups made with contaminated water, and washing of kitchen utensils or other objects with contaminated water cannot be ruled out either. The relatively high coinfection percentage with *G. intestinalis* (20.0% in Atlixco – see Table 6) has already been used as a biomarker indicating high risk of fascioliasis infection by freshwater drinking, because of the significant positive association between *F. hepatica* and this protozoan [5], [50].

Finally, a fascioliasis infection risk related to the drinking of alfalfa juice may neither be neglected. Indeed, it appears as frequent among dietary traditions of children from the Atlixco municipality (Table 3). Involvement of local beverages in human infection is known in areas as Cape Verde [70], [72], and a probable role of alfalfa juice has been highlighted in Peru [73].

### Nitazoxanide treatment of fascioliasis

Nitazoxanide showed an efficacy of 94.0% and 100% after first and second treatment courses, respectively (Table 7). Such a parasitological cure rate is somewhat lower than the 97% obtained in Egyptian children [74] and higher than those obtained in children and adults combined in Egypt (82%) [75] and Peru (49.2%) [76]. Anyway, it should be considered that in Atlixco all liver fluke infections were mild (e.g., low epg) whereas in Peru and Egypt burdens use to be higher [5], [7]. The aforementioned studies were nitazoxanide trials which included patients receiving 500 mg (adults) or 200 mg (children) morning and evening for 6 consecutive days in Egypt [74], [75], and 100 mg (age range 2–3 years), 200 mg (age range 4–11 years) or 500 mg (age higher than 12 years) morning and evening for 7 consecutive days in Peru [76].

It should be emphasized that the only three children still intermittently shedding a low number of *Fasciola* eggs after the first nitazoxanide treatment course, were all of them concomitantly infected by moderate burdens (moderate epg counts) of ascariasis. This may suggest a possible interaction of these large size nematodes with the drug and/or its correct adsorption. Evidence from clinical studies on *A. lumbricoides* infection, primarily in children, shows that ascariasis can be responsible for decreased absortion of nutrients and that infected children may show jejunal mucosal abnormalities which revert to normal after deworming [77].

Treatment results obtained suggest that nitazoxanide may be considered, at least for the chronic stage of fascioliasis, a good alternative to triclabendazole, the drug of choice for human fascioliasis at present [32], [78], [79], mainly in countries where the latter is still not registered but nitazoxanide is since several years, as in Mexico. Nitazoxanide had demonstrated its efficacity against human fascioliasis in a few trials, in Egypt [74], [75] and Peru [76]. However, differences in fasciolid susceptibility to nitazoxanide may exist depending on geographical strains. Thus, no response to nitazoxanide treatment was reported in 24 cases of liver fluke infection in Esmeralda, Camagüey, Cuba [80].

Moreover, its usefulness for the treatment of human cases not responding to triclabendazole [81] is of important additional value, given the spread of the resistance to this drug. However, it should be noted that a triclabendazole-resistant *F. hepatica* infected patient not responding to nitazoxanide treatment has recently been reported in the Netherlands [82]. In animals, triclabendazole resistance was first described in Australia [83], later in Ireland [84], [85], Scotland [86], the Netherlands [87], [88], and Spain [89]. Very recently it has also been found in southern Brazil [90] and Argentina [91]. Up to that moment, triclabendazole resistance only concerned livestock in animal endemic areas, but unfortunately it has very recently been also described in humans [92] in a human highly endemic area such as Cajamarca, Peru [6].

The strategies to minimize the development of resistance include the use of synergistic drug combinations [93], although this approach has the risk of building up multiple drug resistance [88]. Additionally, studies suggest that our understanding of the mechanism of resistance to triclabendazole remains far from complete [94]–[96], so that there is even a knowledge gap regarding its spreading capacity. It is evident that studies for alternative drugs for human use are in urgent need and nitazoxanide appears hence in the frontline.

### Intestinal protozooses and helminths and their treatment

With the exception of the two schools within Atlixco city (Tenextepec, Altavista), in which prevalences were lower, as expected due to its urban location, results of the remaining eight schools indicate that the rural area appears to be homogeneous, indicating a similar insufficiency of hygienic standards throughout.

Prevalence results obtained agree with previous reports indicating that Atlixco municipality should be recognized as one of the areas of the country most affected by intestinal parasitoses [52], [97]. The relatively high infection rates by pathogens such as *G. intestinalis*, *A. lumbricoides* and *T. trichiura*, without forgetting potential *E. histolytica*, pose a public health problem with impact on child development. However, intensities only appear to be low, in contrast to other areas of Mexico where diseases such as hymenolepiasis, ascariasis and trichuriasis have been reported to reach levels of moderate to heavy burdens [41].

For protozooses, nitazoxanide showed high cure rates for all. In infections by *E. histolytica*/*dispar*, a second treatment course was needed for only a very few children (efficacy of 86.8%) (Table 7). This result is very similar to the rates of 81%, 85% and 96% obtained in previous trials [41]–[43]. A similar efficacy (92.6%) was found in infections by *G. intestinalis*, which appears similar to the 94% obtained in Egypt [98], and higher than efficacies (71%, 69% and 64%) obtained in other studies [41]–[43]. The low giardiasis burdens found in Atlixco may explain our results. The maximum efficacy obtained against *B. hominis* with the first treatment course agrees with the very high efficacies (100%, 97%) already observed against this protozoan [41], [43], as well as with its usefulness in cases of persistent diarrhea and enteritis associated with this parasite [99].

Similarly, the efficacy of this drug in treating the few hymenolepiasis cases in Atlixco agrees with results obtained in patients with low burdens by this cestode also in Mexico, such as 97% [41] and 95% [42], which are higher than those of 84% and 85% obtained with moderate burdens [41], [98].

In both ascariasis and trichuriasis, the cure rates obtained were 100% already in the first treatment course (Table 7). Such a high efficacy was also obtained in ascariasis in previous assays [41], [43] when concerning light infections (<2000 eggs/g). In trichuriasis, a 100% efficacy has also been previously obtained [43], but somewhat lower rates ranging between 78% and 89% have also been described [41], [42], [98]. In these two nematodiases, nitazoxanide efficacy has been noted to decrease pronouncedly (down to 48% and 56%, respectively) in infections by moderate to heavy burdens (higher than 2000 eggs/g) [41]. The maximum cure rates obtained in ascariasis and trichuriasis in Atlixco are obviously related to the light burdens present.

With regard to the aforementioned helminthiases, results of trials having demonstrated the usefulness of nitazoxanide as alternative to albendazole (the drug of choice for these helminthiases), in Mexico [100] and also in a human fascioliasis endemic area of Peru [101], are worth mentioning.

In the case of ancylostomatids, all infected children were in need for a second treatment course (Table 7), which contrasts with the 96% cure rate obtained against *A. duodenale* infection in another trial [98]. Given the larval intraorganic migration and the relatively short patent period of 6–7 weeks of these nematodes, treatment failure may be related to tissue migrating larvae at the moment of treatment and perhaps also reinfection of children used to barefooted habits.

The cure rate of 80.9% obtained in oxyuriasis with the first treatment course is similar to the one (80%) already obtained in Mexico before [41], although somewhat lower than those (95%, 100%) found in other assays [43], [98]. Anyway, the easy reinfections in closed environments with soils highly contaminated by eggs (school rooms, family infections, etc.) as well as the selfinfection ways characteristic of this ageohelminthiasis, suggest that the treatment regime for nitazoxanide should not be different from the repeated treatment courses needed for oxyuriasis when applying other nematocides.

Synergistic associations of fascioliasis with other pathogens are believed to be at the base of the high morbidity and mortality rates of children in human fascioliasis hyperendemic areas [45], as the consequence of (i) the pathogenicity of the long-term advanced chronic stage of fascioliasis [15]–[17] and (ii) the immunesuppression induced by the liver fluke throughout the chronic phase [18]. The wide spectrum efficacy of nitazoxanide against fascioliasis and both intestinal protozooses and helminthiases, its high parasitological cure rates, low cost, efficacy similar to albendazole, and usefulness against giardiasis make nitazoxanide a very useful drug for human fascioliasis endemic areas. Indeed, fascioliasis is characterized by presenting high rates of coinfection with other protozooses and helminthiases, among which giardiasis, ascariasis and trichuriasis appear in the forefront [5], [7], [49], [102]. Results obtained in the present study only highlight a potential problem in cases of massive infections by *A. lumbricoides* and *T. trichiura*, for which a somewhat longer treatment course may, in given cases, be applied after previous burden estimation by means of a quantitative diagnostic technique, such as Kato-Katz or any other [32].

## Supporting Information

Checklist S1 STROBE (checklist).
(DOC)
